# Effect of carbonization degree of carbon dots on cytotoxicity and photo-induced toxicity to cells

**DOI:** 10.1016/j.heliyon.2019.e02940

**Published:** 2019-12-05

**Authors:** Neda Esfandiari, Zeinab Bagheri, Hamide Ehtesabi, Zahra Fatahi, Hossein Tavana, Hamid Latifi

**Affiliations:** a Faculty of Life Sciences and Biotechnology, Shahid Beheshti University G.C, Tehran, Iran; b Protein Research Center, Shahid Beheshti University G.C, Tehran, Iran; c Department of Biomedical Engineering, The University of Akron, Akron, OH, 44236, USA; d Laser & Plasma Research Institute, Shahid Beheshti University G.C, Tehran, Iran

**Keywords:** Cell culture, Cell death, Cytotoxicity, Cancer research, Toxicology, Carbonization degree, Carbon dots, Photo-induced toxicity

## Abstract

**Background:**

Pristine carbon dots (CDs) derived from citric acid pyrolysis are used in a variety of biomedical research such as imaging and drug delivery. However, potential cytotoxic effects of pyrolysis temperature on cells is underexplored. To address this need, we studied toxicity of the CDs to breast cancer cells using MTT and LDH assays. In addition, we investigated photo-induced cytotoxicity of the synthesized CDs in a wide concentration range under white light.

**Results:**

Our results suggest little cytotoxicity of the CDs after 24 h exposure of cells. Only the high quantum yield CDs caused a significant toxicity to cells at the highest concentrations of 2.0 and 1.5 mg/ml compared to other CDs at similar concentrations. The synthesized CDs entered the cells without any significant cytotoxicity. The CDs also caused a concentration- and irradiation time-dependent photo-induced cytotoxicity.

**Conclusion:**

The optimization of synthesis conditions from this study may help develop safe and efficient CDs for imaging and drug delivery.

## Teaser

Cell culture; Cell death; Cytotoxicity; Cancer research; Toxicology; Carbonization degree; Carbon dots; Cytotoxicity; Breast cancer cells; Photo-induced toxicity

## 1 Introduction

Carbon is an excellent biocompatible, largely non-toxic, and environmentally friendly compound [1, 2, 3]. Carbon dots (CDs) are zero-dimensional carbon nanomaterials smaller than 10 nm. Due to their unique size [4], chemical and physical properties, water solubility, low production cost, and high photo-stability, carbon dots have received much attention for biomedical applications. Several approaches and materials have been used to prepare CDs with a high photoluminescence (PL) efficiency [5]. These approaches are top-down or bottom-up [6] and include laser ablation [7], acidic oxidation [8], pyrolysis [9], hydrothermal treatments [10, 11], and microwave synthesis [12]. In addition, different materials including chemical and natural products [13] and raw materials such as glycerol, graphite oxide, and citric acid are used to fabricate CDs [14, 15]. Most of the reported research with simple pyrolysis or carbonization of small molecules involved synthesis of high performance CDs as imaging agents. Generally, these methods are economical, simple, and scalable [16]. Carbonization or pyrolysis methods were exploited to fabricate multicolor CDs (blue, green, yellow, and red). CDs have found applications in a various areas including biological sensing [17], bioimaging [18, 19, 20, 21], drug delivery, and photovoltaic devices, although several issues such as time-consuming and tedious synthesis and need for expensive instruments in the production processes remain [22, 23, 24, 25]. Thus, low-cost, rapid, and reliable synthesis of high quality and low cytotoxic CDs in large scales is still highly desirable [26]. Pyrolysis of citric acid, which is a facile bottom-up technique, is a potential approach to address this need [27].

Although nanoparticles have been used in clinical trials and clinically approved for limited applications such as cancer imaging and iron replacement [28], cytotoxicity remains a major barrier against greater clinical translation. CDs hold the promise as potentially safe vehicles for biological applications due to their biocompatibility and low toxicity [29, 30]. CDs mainly enter cells via endocytosis and highly concentrate in the cytoplasm, although low amounts of CDs in the nucleus have also been reported [31]. CDs were successfully used for in vitro imaging of cell transfection and cytotoxicity [32, 33]. CDs with ultra-small size of less than 10 nm are more desirable as drug carriers and for imaging of living cells due to their ability to readily cross the cell membrane [29, 30].

Although cytotoxicity of CDs in different concentrations to various cell lines has been studied, the role of synthesis process on the toxicity of the fabricated CDs is underexplored. To address this knowledge gap, we study the effect of carbonization degree in the synthesis of four different CDs on cytotoxicity, photo-induced toxicity, and cellular uptake under different experimental conditions.

Our results indicate only little cytotoxicity at the highest concentration of CDs to normal and cancer cell lines. Our optimized synthesis may be used as a guide to prepare safe CDs as drug carriers and imaging probes.

## 2 Experimental

### 2.1 Reagents

For synthesis and analysis of CDs, all the chemicals were purchased from Merck and used as received. Ultrapure water was used for diluting samples. Cell culture reagents, including Roswell Park Memorial Institute 1640 medium (RPMI-1640), Dulbecco's Modified Eagle's Medium (DMEM), fetal bovine serum (FBS), penicillin/streptomycin, trypsin/EDTA, and phosphate buffered saline (PBS) were purchased from Gibco. MTT and lactate dehydrogenase cytotoxicity assay kits were obtained from Sigma and Promega, respectively.

### 2.2 Synthesis and characterization of fluorescent CDs

**Figure 1:**
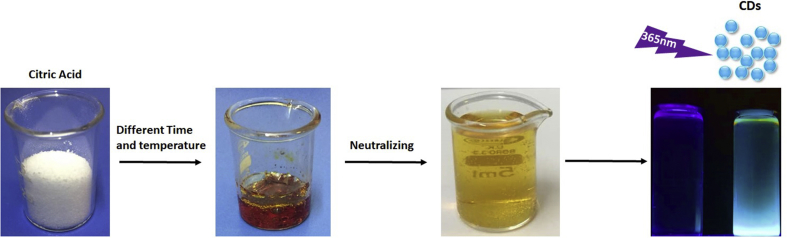
Synthetic production of CDs. Carbon dots with high yield were directly synthesized by pyrolizing citric acid in different time and temperature range. The CDs possess bright blue luminescence.

CDs were prepared by directly pyrolyzing citric acid (Figure 1). Briefly, 1 gr of anhydrous citric acid was heated at specific temperatures for defined incubation times. The liquid changed from colorless to dark brown, implying the formation of CDs. Then, all samples were neutralized using a 0.5 M NaOH solution to a pH of 7.0. Samples were assigned to four groups based on the temperature and incubation time, i.e., 220-20, 200-30, 180- 40, and 160-50, where the first number in each pair shows temperature in degrees Celsius and the second number shows incubation time in minutes. Dynamic light scattering (DLS) was used to measure CDs particle size. Zeta potential of the CDs was determined using ZEN 3600 (Malvern Instruments Co., USA) for different formation samples. The identification of functional groups was done using a Bruker, Tensor27, FT-IR. To confirm morphological characteristics of the particles, scanning transmission electron microscopy (Tecani™ G^2^ F20, FEI Company) was used at 200 kV.

### 2.3 Measurements of fluorescence quantum yield

To determine the quantum yield (QY) of synthesized CDs, quinine sulfate in H_2_SO_4_ (0.1 M, QY = 0.54) at 365 nm was selected as the standard. A low absorbance (<0.05) was used to determine the QY of the samples. The QY of the CDs was calculated from the following equation:

(1) $$QY=\;QY_{R}\;\frac{I}{I_{R}}\;\frac{A_{R}}{A}\frac{n^{2}}{n_{R}^{2}}$$

where I represents measured integrated fluorescent emission intensity, A is the absorbance measured at the excitation wavelength, and n represents the refractive index of the solvent. The subscript R designates the corresponding parameter for a known fluorescent standard [7, 34].

### 2.4 Cell culture and viability analysis

Human breast cancer cell line SKBR3 and normal human breast epithelial cell line MCF-12A were purchased from the Pasteur Institute. The SKBR3 cells were cultured in RPMI-1640 containing 10% (v/v) FBS, 10% (v/v) Pen Strep (50 U/ml penicillin and 50 μg/ml streptomycin). MCF-12A cells were cultured in DMEM supplemented with 10% (v/v) FBS and 1% (v/v) Pen Strep. The cells were seeded into 96-well plates at a density of 1×10^4^ cells/well in 100 μL of medium. After overnight incubation at 37 °C and 5% CO_2_, the medium was replaced with 100 μL fresh complete media (RPMI and DMEM for SKBR3 and MCF-12A, respectively) containing 0.1, 0.2, 0.5, 1.0, 1.5, and 2.0 mg/ml CDs for each formation sample (160-50, 180-40, 200-30, and 220-20). After 24 h of incubation, an aliquot of MTT solution at 5 mg/mL in PBS was added to each well and incubated for 4 h at 37 °C. Then, the content of each well was dissolved by the adding 100 μL of a solubilizing solution for 15 min at room temperature. The absorbance was measured at 570 nm and with a 630 nm reference using a microplate reader (BioTek, ELx 800). Data were collected in triplicates and from four independent parallel experiments. Cell viability was calculated as the ratio of absorbance of sample cells containing CDs to vehicle control cells without any CDs.

### 2.5 LDH assay

SKBR3 and MCF-12A cells were cultured with 0.1, 0.2, 0.5, 1.0, 1.5, and 2.0 mg/ml CDs for each formation sample (160-50, 180-40, 200-30, and 220-20) overnight. Additionally, lysis solution was added to the positive control wells to generate a maximum LDH release control. Then, the supernatants were transferred into a 96-well plate and LDH activity was determined using a standard kit (Promega). The absorbance of supernatants was measured at 490 nm using a microplate reader (BioTek, ELx 800). The cytotoxicity was calculated as the ratio of LDH release from sample cells containing CDs to that from the maximum LDH release vehicle control.

### 2.6 Cellular uptake analysis

SKBR3 cells were cultured in 24-well plates. After 24 h, cells were treated with different formations of CDs (160 -50, 180-40, 200-30, and 220-20) at a 1 mg/ml concentration for 5 h. Cells were washed with PBS and prepared for analysis. The cells were imaged with a fluorescent inverted microscope at 365 nm excitation and 470 nm emission wavelengths.

### 2.7 Photo-induced toxicity analysis

To determine the ability of the CDs to produce cytotoxic effects in cancer cells upon irradiation, 1×10^4^ SKBR3 cells were seeded into 96-well plates in complete RPMI-1640 medium. After 48 h, increasing concentrations (0.1, 0.2, 0.5, 1.0, 1.5, and 2.0 mg/ml) of four CDs formations were added for 24 h. Next, the plates were irradiated using white light for 0 h (as a vehicle control), 1 h, 2 h, and 4 h, followed by incubation at 37 °C and 5% CO_2_ in the dark overnight. On the day of cytotoxicity assay, 5 mg/ml MTT solution in RPMI was added to each well and incubated at 37 °C for 4 h. After removing the medium, DMSO was added. The absorbance was measured at 590 nm after 15 min of incubation. This experiment was repeated three times, each in triplicates.

### 2.8 Statistical analysis

All results were presented as mean ± SEM. Analysis of variance (ANOVA) followed by Tukey post hoc test was performed using SPSS 16.0 statistical package, with p < 0.001 defining statistical significance. Prior to the parametric tests, the normality distribution of data was tested using Kolmogorov-Smirnov and Shapiro-Wilk tests.

## 3 Results and discussion

CDs are valuable materials in biomedicine. Systematic optimization of carbonization degree of CDs is critical to reduce their cytotoxic effects. The objective of this work was to optimize deriving CDs from citric acid pyrolysis and explore their physicochemical properties and toxicity to cells.

### 3.1 Synthesis and characterization of CDs

**Figure 2:**
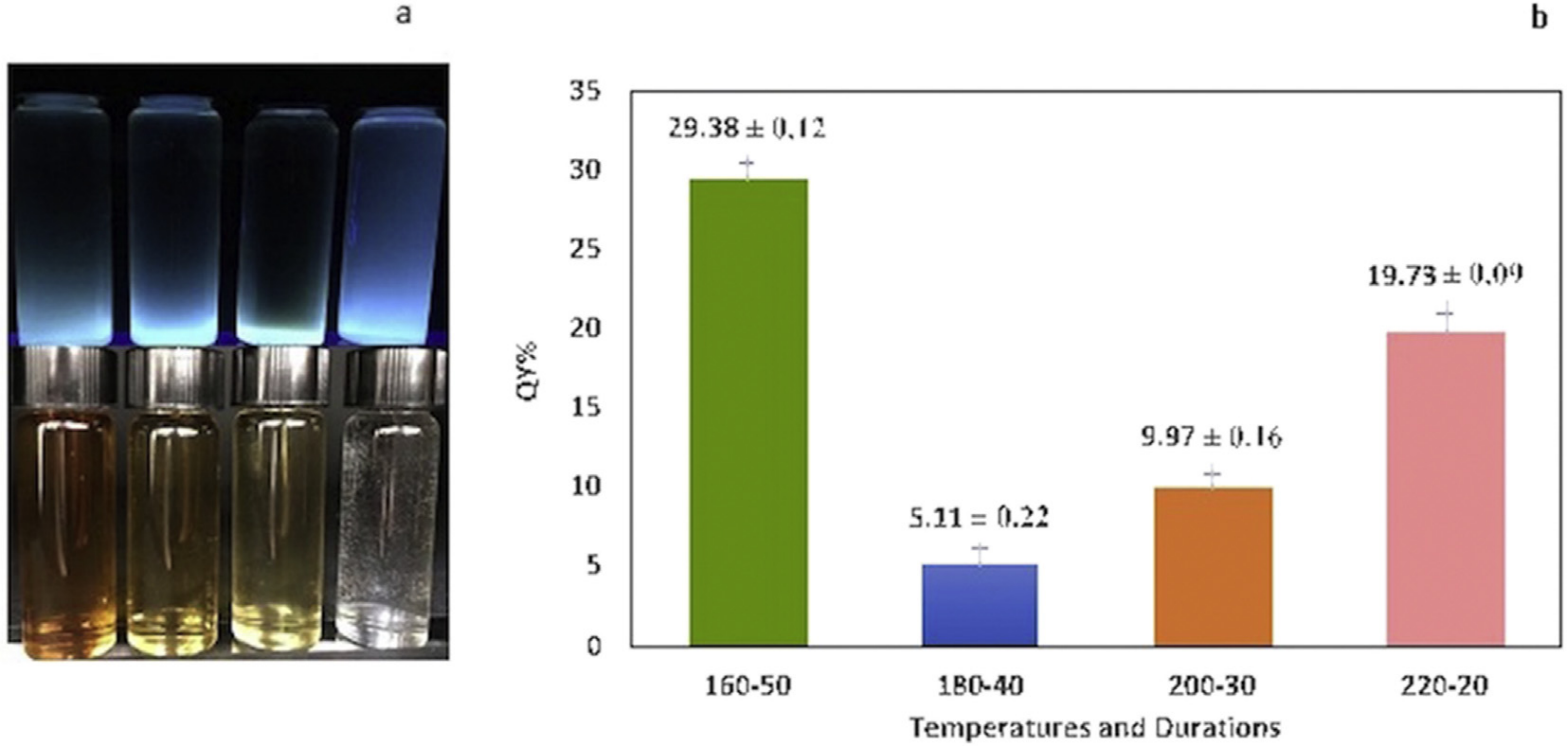
a) Image of synthesized CDs at a wavelength of 365 nm (bottom) and at visible light that shows shades of yellow (top). b) QY of the samples synthesized at different temperatures and durations.

**Figure 3:**
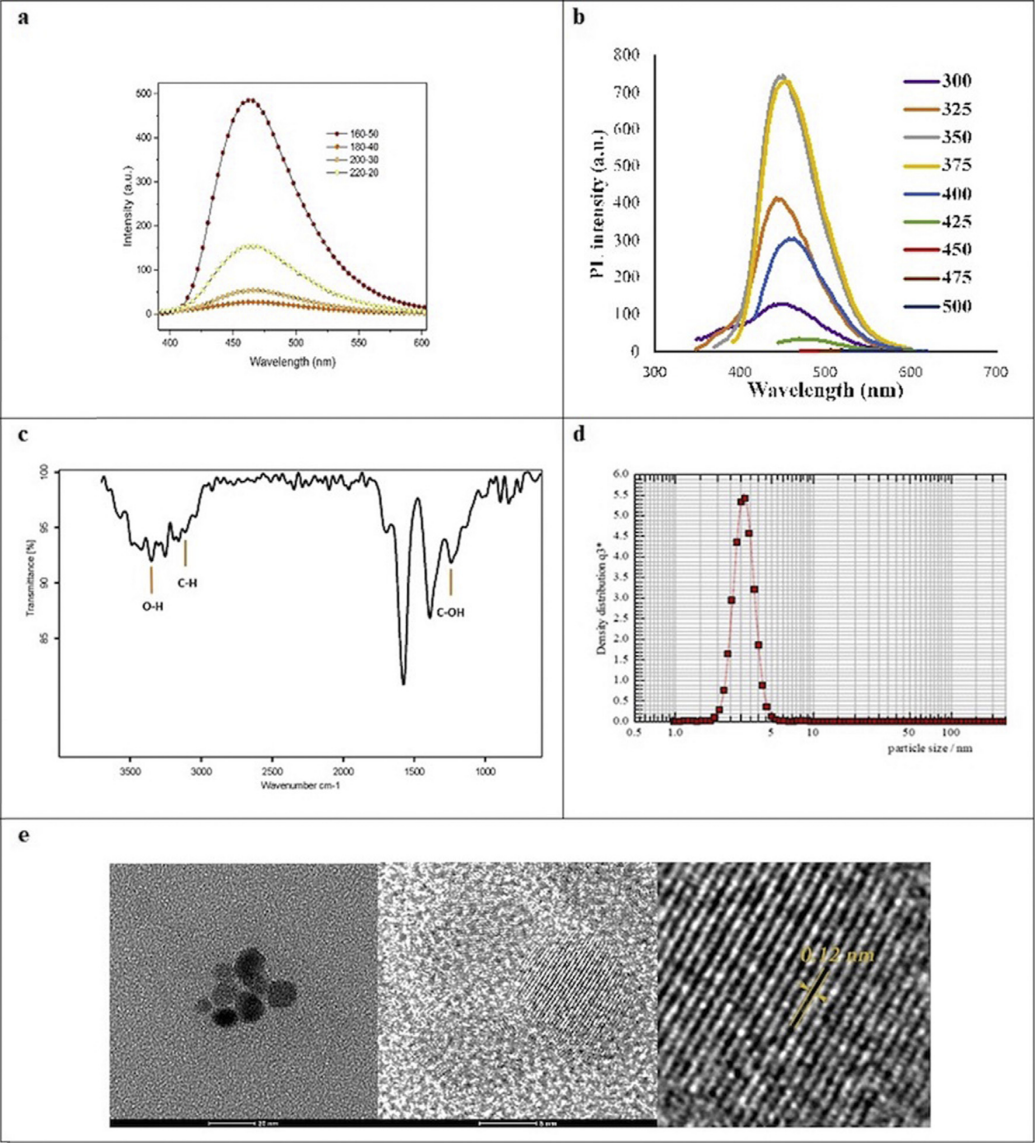
CDs characterization: a) Photoluminescence of samples, b) PL spectra at different excitation wavelength, c) FTIR results d) DLS graph shows particle size distribution for CDs. e) HRTEM image of the CDs shows morphological characteristics of the CDs and their lattice spacing.

Variations in heating time and temperature have major effects on the formation and photoluminescence (PL) properties of CDs [35]. Therefore, we synthesized CDs under four different temperature-time conditions: 220-20, 200-30, 180-40, and 160-50. The CDs after neutralization are shown under visible light and UV irradiation in Figure 2a. We determined the QY of all four samples. The 160-50 sample displayed a strong fluorescence with QY of 29.4% when exposed to UV light at 365 nm (Figure 2b). This QY is significantly higher than that from a previous reports, i.e., ~4–10% [36]. Our measurements showed that the 160-50 sample also had the highest photoluminescent emission (Figure 3a). Furthermore, according to Figure 3b, the emission peaks of the synthesized CDs showed no significant shift at different excitation wavelengths. The highest emission intensity was observed at 350 and 375 nm. Fourier transform infrared (FTIR) spectra in Figure 3c indicated absorption of stretching vibration O–H, C–H, and C–OH groups [35]. To determine the size distribution of CDs dynamic light scattering (DLS) was employed [37, 38, 39, 40]. The result showed that CDs were under 5 nm in diameter (Figure 3d), in agreement with previous reports that showed the size distribution of carbon dots was under 10nm [41, 42]. The high-resolution TEM (HRTEM) analysis (Figure 3e) confirms that these monodisperse nanoparticles are mostly spherical and lattice finger with the d-spacing of 0.12 corresponding to the (102) lattice space of graphitic carbon [43].

**Figure 4:**
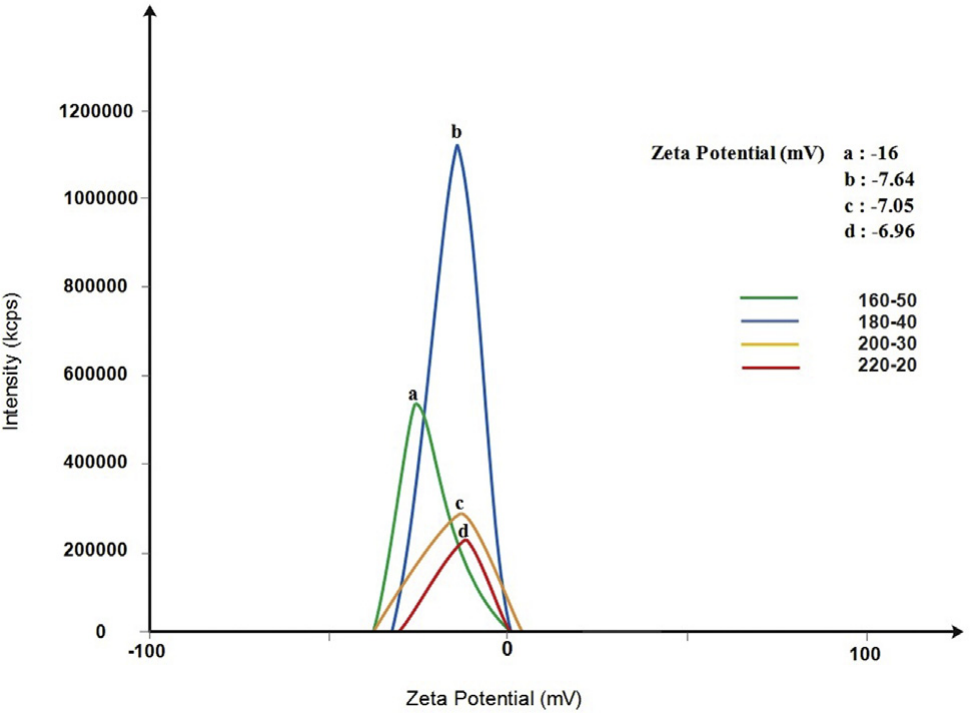
Zeta potential of the different CDs: a) 160-50, b) 180-40, c) 200-30, and d) 220-20. All four CDs have a negative surface charge.

To determine the surface charge of the particles, we measured the zeta potential of the CDs. All four samples displayed negative zeta potential values in the range of -6.96 mv for the 220-20 samples to -16.0 mv for the 160-50 samples (Figure 4). Decreasing the temperature and increasing the incubation time increased the negative charge on the surface of the CDs. Interestingly, while changing temperature-time from 220-20 to 200-30 and to 180-40 had small effects and changed the zeta potential by 1.23% and 9.8%, respectively, the 160-50 samples showed a 130% increase in negative surface charges.

**Figure 5:**
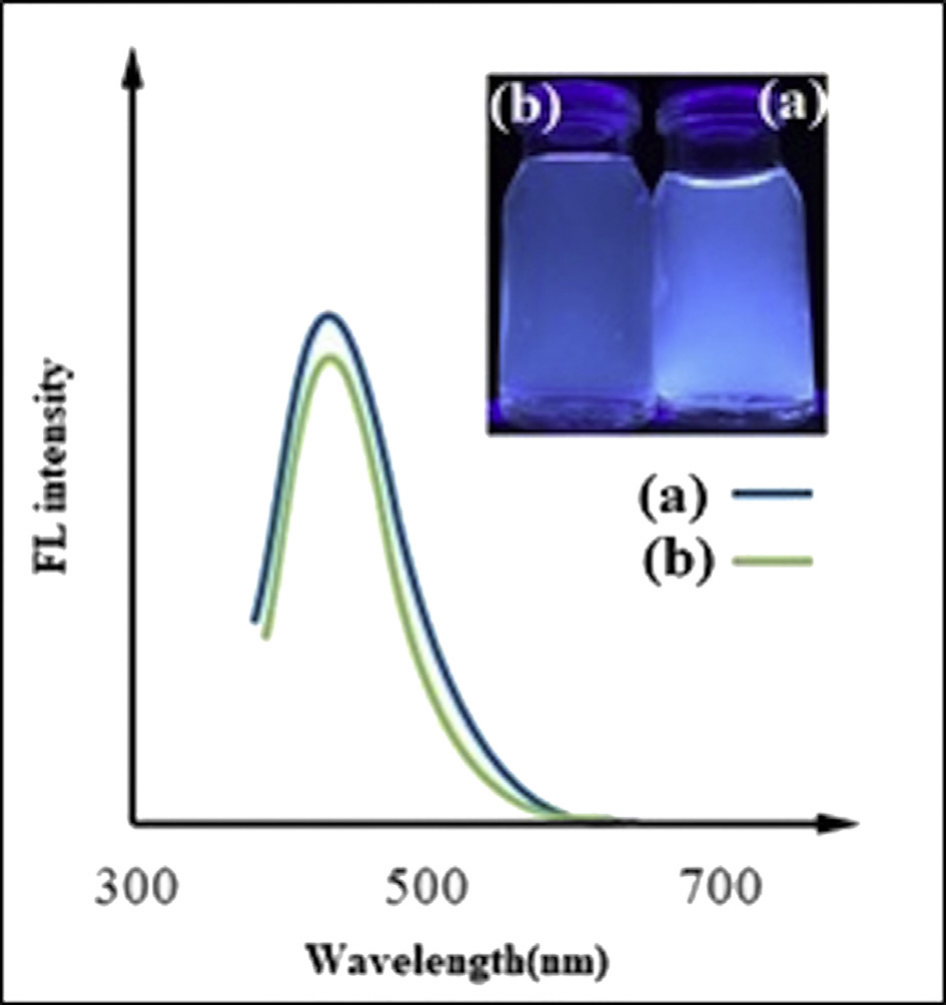
Comparison between fluorescent intensity of fresh CDs and CDs synthesized 6 months ago: CDs solution under UV light and fluorescent spectra of both a) fresh synthesized CDs and b) CDs prepared 6 months ago.

To evaluate the long-term stability of CDs, fluorescent intensity of an aqueous solution of CDs was measured 6 months after preparing them. CDs completely retained their fluorescent property (Figure 5). Photo-stability studies suggest that CDs have a great potential in biological applications such as cell imaging, biosensing, and drug/gene delivery [44].

### 3.2 Cytotoxicity and cellular uptake

**Figure 6:**
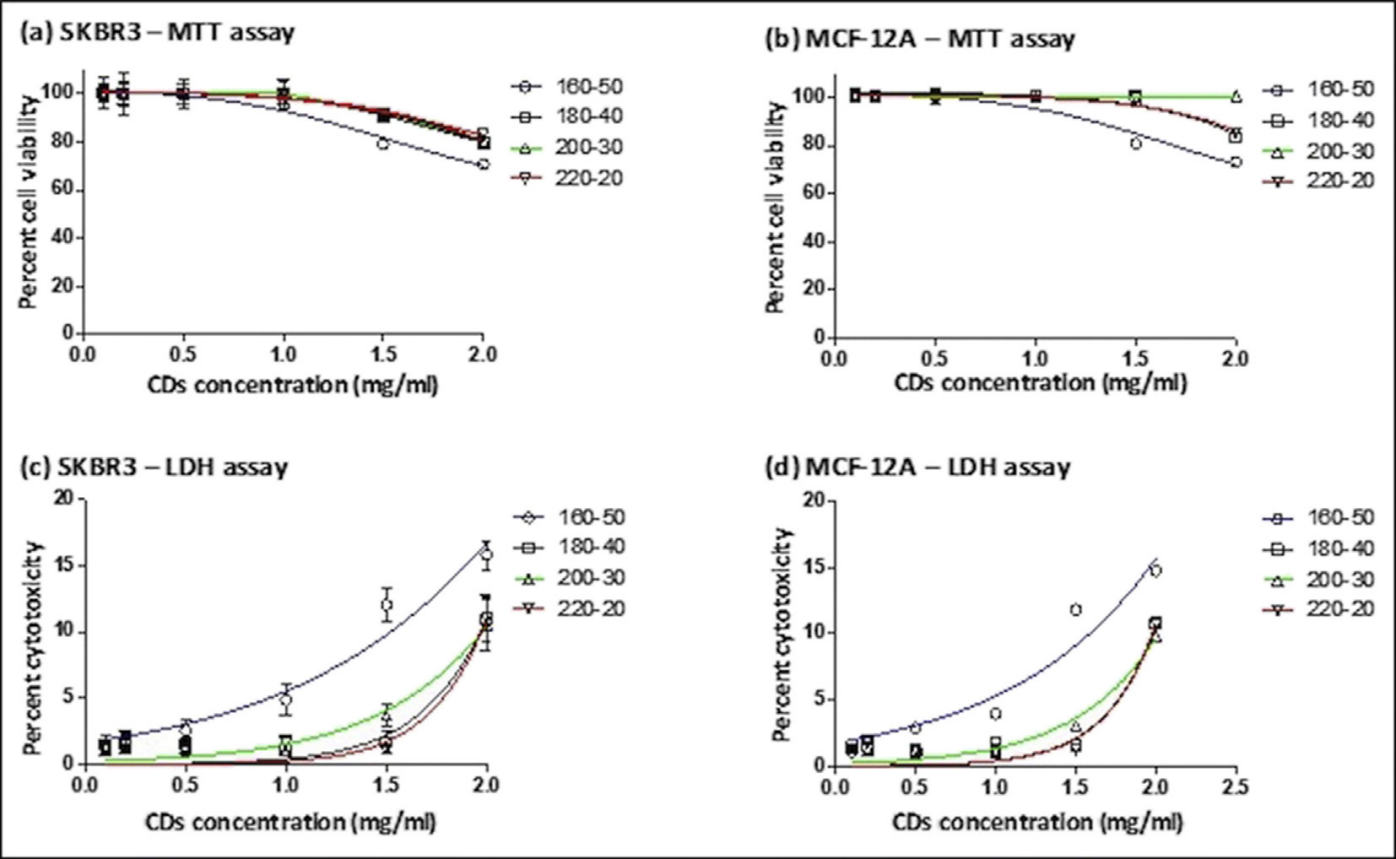
In vitro cytotoxicity of four different formations of CDs with (a) SKBR3 cells and (b) MCF-12A cells using MTT assay, and (c) SKBR3 cells and (d) MCF-12A cells using LDH assay. Asterisks indicate difference between the 160-50 (2 mg/ml) and the other CDs. Quantities represent mean ± SEM (*p ≤ 0.001, one-way ANOVA).

We conducted a cytotoxicity study at different concentrations of CDs (0.1–2.0 mg/ml) with each formation sample and evaluated the responses of breast cancer SKBR3 and normal MCF-12A cells using MTT. Figure 6a-b shows that the viability of SKBR3 and MCF-12A cells treated with different formations of CDs for 24 h was not affected at concentrations of ≤1.0 mg/ml. At higher concentrations, the cell viability decreased dose dependently. The 160-50 sample caused the largest cytotoxicity and significantly (p ≤ 0.001) reduced the cell viability to 78% and 70% at 1.5 and 2.0 mg/ml, respectively. With the other three formations, cell viability was ~92% and ~81% at these two concentrations. This result suggests using CDs at concentrations of ≤1.0 mg/ml to avoid toxicity to cells.

Next, we performed an LDH assay to investigate potential membrane damage to SKBR3 and MCF-12A cells by CDs after 24 h of incubation. LDH is a soluble cytoplasmic enzyme and is released into culture medium upon damage to plasma membrane. Consistent with the cell viability study, the 160-50 CDs resulted in a significant membrane damage at concentrations of 1.0, 1.5, and 2.0 mg/ml, whereas the other three formations compromised membrane integrity only at 2.0 mg/ml (p ≤ 0.001). The largest LDH release in SKBR3 cells at 2.0 mg/ml of 160-50, 180-40, 200-30, and 220-20 formations were 15.82% ± 0.35, 11.03% ± 0.57, 10.56% ± 0.61, 11.03 ± 0.54, respectively, compared to 1.00% ± 0.59 when the control formation was used (Figure 6c). In MCF-12A cells, the largest LDH release at 2.0 mg/ml of 160-50, 180-40, 200-30, and 220-20 formations were 14.76% ± 0.20, 10.81% ± 0.28, 9.87% ± 0.11, 10.73% ± 0.23, respectively, compared to 1.05% ± 0.05 when the control formation was used (Figure 6d). Therefore, even at high concentrations, these CDs exhibited little toxicity toward cells compared to other reports [45, 46]. Greater toxicity of the 160-50 sample than the other three formations is most likely due to its significantly larger zeta potential [47, 48], indicating uptake of the 160-50 CDs by SKBR3 and MCF-12A cells than the other formations.

**Figure 7:**
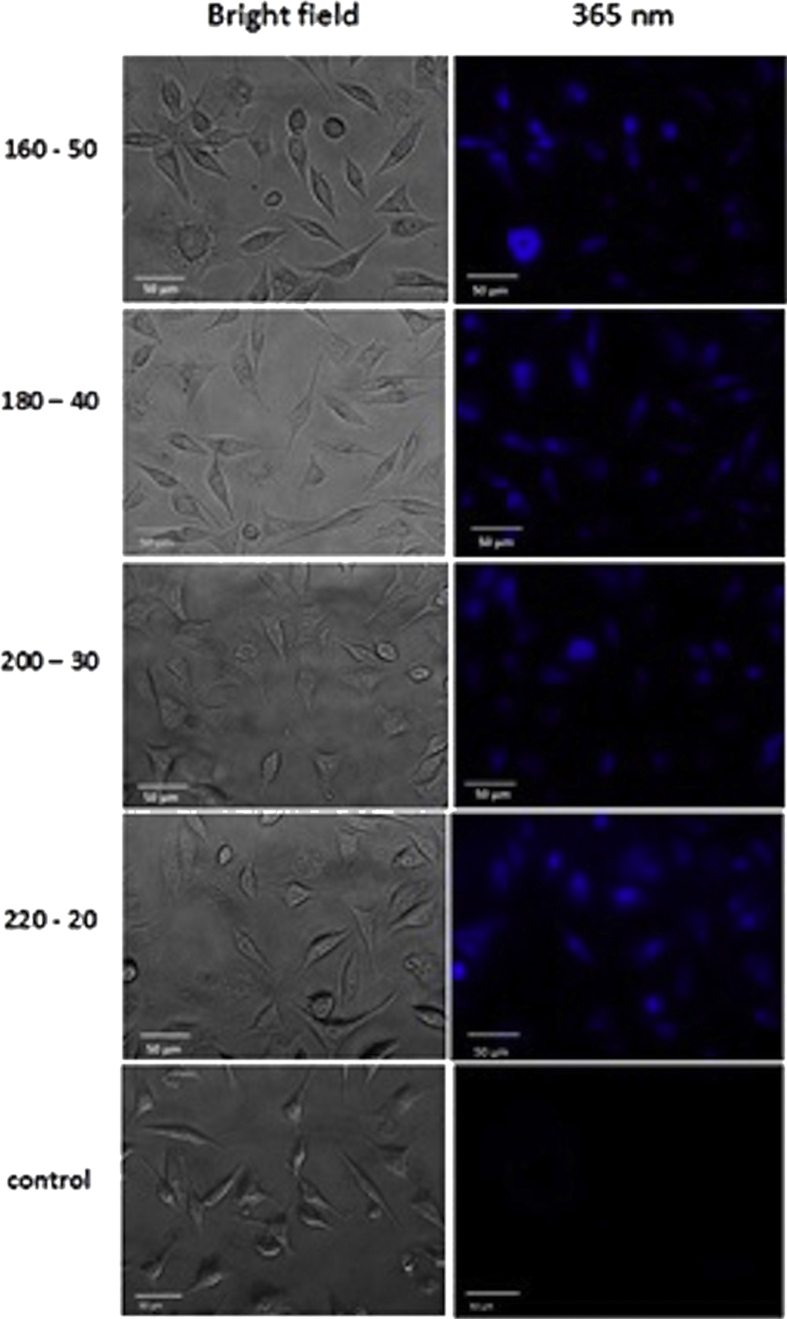
Phase and fluorescent images of SKBR3 cells incubated with different formations of CDs (160-50, 180-40, 200-30, and 220-20) under bright field and excitation of 365 nm wavelength.

As shown in Figure 7, the measured fluorescence intensity from SKBR3 cells treated with different CDs was not dependent on the specific formations. The vehicle control cells (without CDs) did not show any fluorescence emission. Cells treated with CDs had comparable morphology to the vehicle control cells. The different formation of CDs did not have significantly different cellular uptake. Therefore, the extent of uptake of different CDs did not cause differences in their cytotoxicity [49].

### 3.3 Photo-induced cytotoxicity

**Figure 8:**
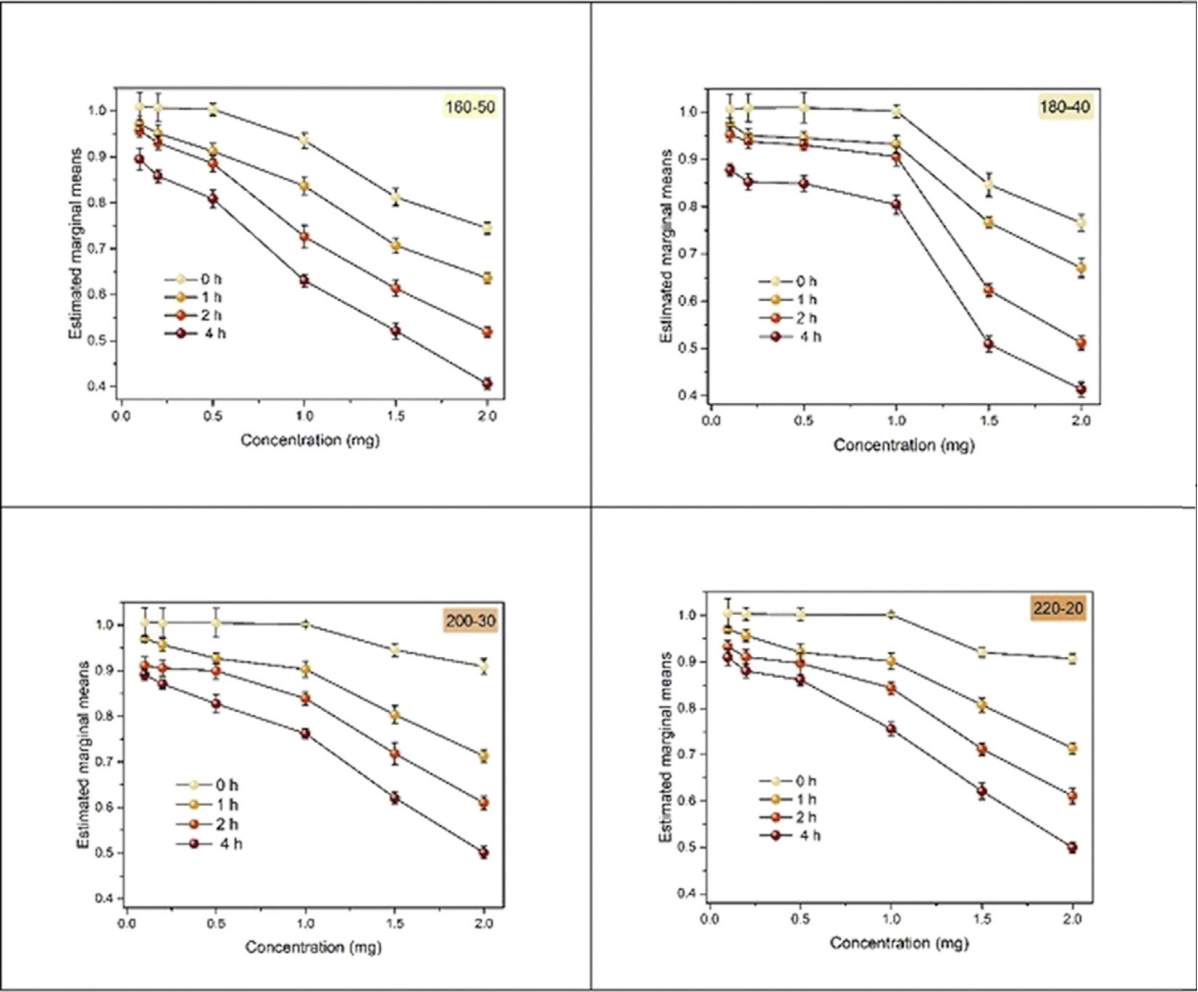
Photo-induced toxicity of CDs. The average percentages of cell viability SKBR3 cells in different samples 160-50, 180-40, 200-30, 220-20 and concentrations (0.1–2.0 mg/ml) and irradiation times (0 h, 1 h, 2 h, and 4 h). The estimated marginal mean is the mean value of a factor averaged across other levels of the factors (P ≤ 0.001, one-way ANOVA).

Next, we used MTT assay to determine whether the CDs are cytotoxic to SKBR3 cells upon irradiation. We irradiated the samples with white light for 0 h (control), 1 h, 2 h, and 4 h, and then kept them in the dark. Based on the variation for sample, even the small differences between the results for 1 h and 2 h in Figure 8 became significant at a level of 0.05.

Thus, one may consider the linear model

(2) $${\text{V}}_{\text{i,j,k}}\;=\;\text{μ}+\;{\text{T}}_{\text{i}}\;+\;{\text{C}}_{\text{j}}\;+\;\left(\text{TC}\right){\;}_{\text{i,j}}\;+\;{\text{ε}}_{\text{i,j,k}}\text{,}$$

for i = 1,...,4, j = 1,...6, k = 1,...,9, In this model, V_{i,j,k} is the rate of survival of the cell in our kth experimental observation when the time factor is set on its ith level and the concentration is set on its jth level. Here, T_i and C_j are respectively the effects of the ith level of time on the observed value of survival rate. Note that there are four levels of time, i.e., 0, 1, 2, and 4 h, and six levels of concentration, i.e., 0.125, 0.25, 0.5, 1, 1.5, and 2 mg/ml. In this model, (TC)_{i,j} is the joint effect of variable time and concentration on the response variable, V. Only for the sake of completeness, we consider a possible interaction effect between time and concentration on the rate of survival at the first step. In this model, ε represents the random error of each experiment, which is a Gaussian white noise. Under some mild assumptions on the ε, firstly this model as a simple ANOVA allows us to test whether the two factors (time and concentration) do significantly affect the rate of survival of cells in our experiments. If so, the post-hoc test of this ANOVA model returns the importance level of each factor on the response variable V. It is worth mentioning that μ is the global mean of survival rate of cells irrespective of time and concentration. As we expected, the ANOVA test indicated that there was no significant evidence to support an interaction effect between time and concentration, i.e.,$H_{0}:{\left(TC\right)}_{i,j}=0$ . Furthermore, increasing irradiation time from 1 h to 4 h showed a noticeable difference in viability of cells (Figure 8). These results suggest that the CDs upon irradiation significantly increased cytotoxicity compare to the vehicle control (P ≤ 0.001).

## 4 Conclusion

We investigated the impact of carbonization degree in the synthesis of four different CDs on cytotoxicity, photo-induced toxicity, and cellular uptake under different experimental conditions. We synthesized carbon dots (CDs) through thermal decomposition of citric acid at different temperatures and time durations and characterized their size, surface charge, quantum yield, and toxicity to cells. The CDs were smaller than 5 nm with a negative surface charge and showed a significantly higher quantum yield than those reported previously, especially with the CDs fabricated at 160 °C for 50 min (160-50 formation). Our analysis of cytotoxicity and photo-induced toxicity of CDs demonstrated their biocompatibility. Except for the 160-50 formation sample that showed a toxicity of ~20–30% to breast cell lines at the highest two concentrations used, other formation samples were non-toxic. Our results demonstrated that this effect was independent of cellular uptake of CDs as endocytosis of all four formation samples was similar. Overall, this study demonstrated that small changes in the synthesis conditions can have significant effects on properties of CDs. Our findings may facilitate the development of safe and biocompatible CDs for imaging and drug delivery systems.

## Declarations

### Author contribution statement

Neda Esfandiari: Conceived and designed the experiments; Performed the experiments; Analyzed and interpreted the data; Contributed reagents, materials, analysis tools or data; Wrote the paper.

Zeinab Bagheri, Hamide Ehtesabi: Conceived and designed the experiments.

Zahra Fatahi: Performed the experiments.

Hossein Tavana: Analyzed and interpreted the data; Wrote the paper.

Hamid Latifi: Contributed reagents, materials, analysis tools or data.

### Funding statement

Neda Esfandiari was in part supported by Shahid Beheshti University.

### Competing interest statement

The authors declare no conflict of interest.

### Additional information

No additional information is available for this paper.
